# Mobile Colistin Resistance Gene *mcr*-*1* Detected on an IncI2 Plasmid in *Salmonella* Typhimurium Sequence Type 19 from a Healthy Pig in South Korea

**DOI:** 10.3390/microorganisms9020398

**Published:** 2021-02-15

**Authors:** Dong Chan Moon, Su-Jeong Kim, Abraham Fikru Mechesso, Hee Young Kang, Hyun-Ju Song, Ji-Hyun Choi, Soon-Seek Yoon, Suk-Kyung Lim

**Affiliations:** Bacterial Disease Division, Animal and Plant Quarantine Agency, 177 Hyeksin 8-ro, Gimcheon-si 39660, Korea; ansehdcks@korea.kr (D.C.M.); kimsujeong27@gmail.com (S.-J.K.); abrahamf@korea.kr (A.F.M.); kanghy7734@korea.kr (H.Y.K.); shj0211@korea.kr (H.-J.S.); wlgus01@korea.kr (J.-H.C.); yoonss24@korea.kr (S.-S.Y.)

**Keywords:** colistin resistance, *mcr-1*, pig, plasmid, *Salmonella* Typhimurium

## Abstract

Colistin is considered the last resort for the treatment of multi-drug resistant Gram-negative bacterial infections. We studied colistin resistance and the *mcr-1* gene carriage in *Salmonella* isolates recovered from food animals in South Korea between 2010 and 2018. Colistin resistance was found in 277 isolates, predominantly in *Salmonella* Enteritidis (57.1%) and *Salmonella* Gallinarum (41.9%). However, the *mcr-1* gene was identified in only one colistin-resistant *Salmonella* Typhimurium (MIC = 16 µg/mL) isolated from a healthy pig. The *mcr-1* carrying isolate presented additional resistance to multiple antimicrobials. The strain belonged to sequence type (ST)19 and carried various virulence factor genes that are associated with adhesion and invasion of *Salmonella* into intestinal epithelial cells, as well as its survival in macrophages. The *mcr-1* gene was identified on an IncI2 plasmid and it was also transferred to the *E*. *coli* J53 recipient strain. The *mcr*-*1*-carrying plasmid (pK18JST013) in this study was closely related to that previously reported in *S*. Indiana (pCFSA664-3) from chicken in China. This is the first report of *mcr-1* carrying *S*. Typhimurium in South Korea. The finding indicates the importance of regular screening for the presence of the *mcr*-*1* gene in *S*. Typhimurium in food animals to prevent the spread to humans.

## 1. Introduction

Non-typhoidal *Salmonella* serovars are common causes of foodborne diseases, especially in the elderly, children, and immunocompromised individuals [1]. Non-typhoidal *Salmonella*-associated enterocolitis was associated with 95 million illnesses and 50,000 deaths globally in 2017, and most of the cases were foodborne [2]. In South Korea (Korea), *Salmonella* was detected in about 8% of gastroenteritis cases in 2017 [3]. Contaminated food products from food animals are the most common sources of human infections [1].

Colistin belongs to the group of polymyxins and has been used for the treatment and prevention of gut infections in food animals particularly poultry and pigs. In humans, although their parenteral usage has been limited because of concerns of toxicity, it is the last resort treatment for severe infections caused by multi-drug resistant (MDR) Gram-negative bacteria, particularly carbapenem-resistant *Enterobacteriaceae* [4]. Colistin use in animals has promoted the selection and transmission of *mcr*-harboring strains [5]. The *mcr* genes encode phosphoethanolamine transferases that modify the lipopolysaccharides of the outer membrane in Gram-negative bacteria, which leads to reduced susceptibility or resistance to colistin [5].

Since the first report of the *mcr*-*1* gene in *Escherichia coli* in China in 2016 [6], the *MCR*-family genes have been detected in several other *Enterobacteriaceae* in many countries [7]. The *mcr-1* and *mcr-9* are the most widely disseminated MCR-family genes, being identified in 40 countries across six continents; *mcr-3* and *mcr-5* are the next most widely spread genes. The remaining MCR-family genes are disseminated across small areas [7]. In Korea, *mcr-1*, *mcr-3*, and *mcr-9* genes were identified in *Enterobacteriaceae* isolated mainly from food animals [8,9,10].

Several studies have identified the *mcr*-*1* harboring *Salmonella* from patients, food and companion animals, and food of animal origin in Asia [11,12,13,14], Europe [15,16], and South America [17,18]. In Korea, however, this gene has been detected only in *E*. *coli* and *Enterobacter aerogenes* isolated from humans, food and companion animals, and fresh vegetables [8,19,20,21]. The *mcr*-*1* gene was so far associated with diverse plasmids belonging to the IncI2, IncHI1, IncHI2, IncP1, IncX4, IncFII, and IncY replicon types. Among them, IncHI2 and IncI2 plasmids were the most common replicons in *Salmonella* spp [22]. In view of the concerning horizontal spread of the *mcr*-*1* gene among *Enterobacteriaceae* in food animals and its significant public health impacts, we performed a retrospective evaluation of colistin resistance and investigated the *mcr*-*1*-carrying plasmid in *Salmonella* isolated from food animals between 2010 and 2018 in Korea.

## 2. Materials and Methods

### 2.1. Identification of Colistin-Resistant and mcr-1 Carrying Salmonella Serotypes

Recently, we identified and serotyped 3018 *Salmonella* isolates recovered from cattle, pigs, and chickens throughout Korea from 2010 to 2018. Isolates were obtained from 16 laboratories/centers participating in the Korean Veterinary Antimicrobial Resistance Monitoring System [23]. In this study, we performed a retrospective evaluation of the colistin susceptibility profiles of these isolates. The minimum inhibitory concentration (MIC) of colistin was determined by the broth microdilution method using Sensitire panel KRNV5F (Trek Diagnostic Systems, Cleveland, OH, USA), following the manufacturer’s instructions. Additionally, multiplex-polymerase chain reaction (mPCR) analysis was performed to determine the *mcr* gene (*mcr*-*1* to *mcr*-*9*) carriage of colistin-resistant serotypes, as described previously [24,25].

### 2.2. Characterization of mcr-1-Carrying Isolate

We investigated the susceptibility profiles of the *mcr-1*-carrying isolate as described above. The following antimicrobials were studied: amoxicillin/clavulanic acid (2/1–32/16 µg/mL), ampicillin (2–64 µg/mL), cefepime (0.25–16 µg/mL), cefoxitin (1–32 µg/mL), ceftazidime (1–64 µg/mL), ceftiofur (0.5–8 µg/mL), chloramphenicol (2–64 µg/mL), ciprofloxacin (0.12–16 µg/mL), colistin (2–16 µg/mL), gentamicin (1–64 µg/mL), meropenem (0.25–4 µg/mL), nalidixic acid (2–128 µg/mL), streptomycin (16–128 µg/mL), sulfisoxazole (16–256 µg/mL), tetracycline (2–128 µg/mL), and trimethoprim/sulfamethoxazole (0.12/2.38–4/76 µg/mL). The MIC values were interpreted according to the Clinical and Laboratory Standards Institute [26] and the European Committee on Antimicrobial Susceptibility Testing [27] guidelines.

Multilocus sequence typing was performed as described previously [28]. Sequence type (ST) was assigned using the multilocus sequence typing website for *Salmonella* (http://pubmlst.org/organisms/salmonella-spp). Plasmid replicon typing was performed using a PCR-based replicon typing kit (Diatheva, Fano, Italy). We investigated the presence of 30 major plasmid incompatibility groups circulating among *Enterobacteriaceae*: IncHI1, HI2, I1, I2, X1, X2, X3, X4, L, M, N, FIA, FIB, FIC, FII, FIIS, FIIK, FIB KN, FIB, KQ, W, Y, P1, A/C, T, K, U, R, B/O, HIB-M, and FIB-M. Additionally, a mPCR assay (iNtRON Biotechnology, Seongnam, South Korea) was performed to detect virulence factor genes that enable *Salmonella* to reach the systemic circulation and increase its infectivity (*cdtB*, *invA*, *iroN, lpfC, msgA*, *orgA, pagC*, *pefA, prgH*, *sifA, sipB*, *sitC*, *sopB, spaN*, *spiA*, *spvB*, and *tolC*), as described previously [29].

Conjugation was performed by the filter mating method using azide-resistant *E. coli* J53 as the recipient strain, as described by Na et al. [30]. Putative transconjugants were selected on a Muller–Hinton agar plate supplemented with sodium azide (150 µg/mL) and colistin (2 μg/mL). The transconjugants were confirmed by PCR detection of the *mcr-1* gene, as described above. Transfer frequency was calculated based on the number of transconjugants obtained per donor and conjugation efficiency is explained as mean ± standard deviation of triplicate experiments. Additionally, the antimicrobial susceptibility profile of the transconjugants was determined as described above.

### 2.3. Whole-Genome Sequencing and Annotation

Genomic DNA was extracted and purified using MG genomic DNA purification kit, according to the manufacturer’s instructions (MGmed, Seoul, Korea). For PacBio RS II (Pacific Biosciences, Menlo Park, CA, USA) sequencing, 8 µg of input genomic DNA was used for 20-kb library preparation. The library insert sizes were optimal; genomic DNA was sheared with g-TUBE (Covaris Inc., Woburn, MA, USA) and purified using AMPure PB magnetic beads (Beckman Coulter Inc., Brea, CA, USA) if the apparent size was >40 kb. We sequenced the plasmid genome to 149× depth using the PacBio Sequel platform. De novo assembly and consensus polishing were performed using the Hierarchical Genome Assembly Process (HGAP) 2 package contained in the SMRT version 2.3.0 software. Assembly data were then circularized using circulator 1.4.0. Glimmer 3 [31] was used to predict genes, and annotation was done using a homology search based on the Clusters of Orthologous Groups (COG) database.

The sequences of the *mcr-1*-carrying plasmid in this study (pK18JST013, GenBank accession number. CP065423) was compared with those of previously reported plasmids (Table S1). Briefly, nucleotide sequences of *mcr-1* carrying plasmids were downloaded from the GenBank nucleotide database. As the sequences have different starting points, sequences were rotated for accurate alignment so that the start sites of sequences were set as RepA using GAMOLA2 [32]. The sequence of each plasmid was aligned using Blastn (v 2.8.1) and compared using EasyFig (v.2.2.3) [33]. Besides that, average nucleotide identity (ANI) values were calculated with pairwise genome alignment of sequences by using the ANI-blast method implemented in PYANI (v.0.2.9) [34] and the phylogenetic tree is reconstructed based on the ANI values.

## 3. Results and Discussion

The overall proportion of colistin-resistant *Salmonella* strains (9.2%, 277/3018) in this study was slightly higher than previous reports from 11 European countries (5.3%, 92/1774) [35], Japan (1.2%, 1/82) [36], and Korea (1%, 1/100) [37] (Table 1). However, it was lower than the finding of Figueiredo et al. [15] (14.3%, 37/258) from retail meat in Portugal. The majority of colistin-resistant strains (98.9%, 274/277) belonged to the *Salmonella* serogroup D (*Salmonella* Enteritidis (57.7%) and *Salmonella* Gallinarum (42.3%)) (Table S2). Indeed, colistin resistance was observed in 49.2% of *S*. Enteritidis and 92.8% of *S*. Gallinarum isolated from chickens. The only colistin-resistant isolate in serogroup B belonged to *Salmonella* Typhimurium (Isolate no. 18-A02-013), while serogroup(s) of the two colistin-resistant strains were unidentified. The colistin-resistant (MIC = 16 µg/mL) *S.* Typhimurium was recovered from a healthy pig from Jeju in 2018. The high colistin resistance rate in *S*. Enteritidis and *S*. Gallinarum isolated from chickens in our study could be due to the intrinsic colistin resistance in these serotypes [38], which is related to the presence and composition of O antigens [39].

Among the colistin-resistant *Salmonella* strains (*n* = 277), we detected the *mcr-1* gene in the only (0.4%) *S.* Typhimurium strain. In agreement with this study, many researchers have reported a very low prevalence of *mcr*-*1* gene in *Salmonella* strains isolated from food animals and humans in other Asian countries (0.3%–2%) [40,41] and Europe (0.1%–3.1%) [15,35,42]. In contrast, Yi et al. [14] (14.8%) and Ma et al. [43] (11.4%) have found a relatively higher percentage of *mcr*-*1* carriage in *Salmonella* isolated from food animals and humans in China. Previous studies have also detected other MCR-family genes (*mcr*-*2* to *mcr*-*5* and *mcr*-*9*) in *Salmonella spp* isolated from different sources [44,45]. Our result suggests that healthy pigs are a matter of concern in terms of transmission of *mcr*-*1*-carrying *Salmonella* to humans through the food chain. The differences in the number of tested isolates and resistance detection methods should be considered while comparing and contrasting findings from other studies.

The *mcr-1*-carrying *S.* Typhimurium in this study (18-A02-013) presented additional resistance to multiple antimicrobials, including resistance to ampicillin, chloramphenicol, streptomycin, sulfisoxazole, and tetracycline (Table 2). This multidrug resistance pattern is characteristic of the European *S*. Typhimurium clones [46,47] and it is strongly associated with pork [48]. Multiple resistance determinants could co-exist with the *mcr*-*1* gene in the same plasmid, and this can generate resistance to multiple antimicrobials [13,49]. Our *S*. Typhimurium strain belonged to sequence type (ST) 19. Consistently, *mcr*-*1*-carrying *S*. Typhimurium ST19 was identified from patients and retail meat in Brazil [17], China [49], and Denmark [42]. Similarly, Suh and Song [50] have identified *S*. Typhimurium ST19, although susceptible to colistin, from swine carcasses in Korea. *S*. Typhimurium ST19 is globally distributed and associated with outbreaks of human gastroenteritis, mainly in Europe and the US [25,51,52]. Additionally, the *mcr-1*-carrying *S.* Typhimurium identified in this study carried a large repertoire of virulence factor genes (Table 2). Except for *iroN* and *sitC*, all the detected virulence factor genes encode products that are related to host cell adhesion, invasion, and intracellular survival [29]. The *iroN* and *sitC* genes were related to other traits thought to be important in *Salmonella* pathogenesis, such as iron acquisition [29,53]. Leite et al. [52] also reported on *mcr*-*9*-carrying *S*. Typhimurium ST19 strain carrying a plethora of virulence factors associated with *Salmonella* adhesion, stress adaptation, immune evasion, and invasion. Thus, the association of the *mcr*-*1* and virulence factor genes with one of the most widely distributed *Salmonella* strains isolated from pig presented a potential risk to public health.

The association of the *mcr-1* gene with different plasmids is vital for its dissemination into various hosts. The *mcr*-*1* gene in *S*. Typhimurium strain isolated in this study belonged to the IncI2 plasmid, a finding consistent with those of Torpdah et al. [42] in Denmark and Lu et al. [54] in China. Similarly, other studies in China [40] and Great Britain [55] have reported the link between the *mcr-1* gene and the IncI2 plasmid in *S*. Typhimurium isolated from patients. The *mcr*-*1*-carrying plasmids are highly stable in bacteria even in the absence of polymyxin selection pressure. Thus, the existence of the *mcr-1* gene embedded into the IncI2 plasmid is a huge concern, because it facilitates the transfer of the *mcr*-*1* gene to other bacterial pathogens of animal and human origin, including commensals [6,55].

The conjugation efficiency of the *mcr*-*1*-carrying IncI2 plasmid in this study (3.0 ± 0.8 × 10^−3^ ) was higher than those of the *mcr*-*1*-carrying IncI2 plasmids reported by Anjum et al. [55] (between 10^−7^ and 10^−9^). However, it was low compared to some of the *mcr*-*1*-carrying IncI2 plasmids reported by Lu et al. [54]. The IncI2 plasmids, which have a broad host range, are commonly associated with the acquisition and transfer of new antibiotic resistance genes. Additionally, they are known to adapt to new bacterial hosts [56].

**Figure 1:**
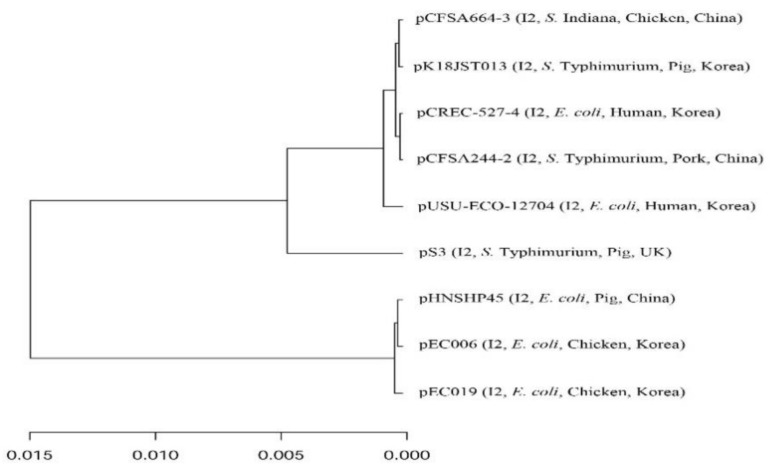
Average nucleotide identity analysis was performed using the ANI-blast method implemented in PYANI (v.0.2.9) and the tree was generated based on the ANI values. The horizontal lines represent the 95% threshold value. The scale bar represents sequence divergence, i.e., the percentage of nucleotide substitution rate over the length of the genome. Detailed information on the sources and characteristics of the compared plasmids is presented in Table S1.

**Figure 2:**
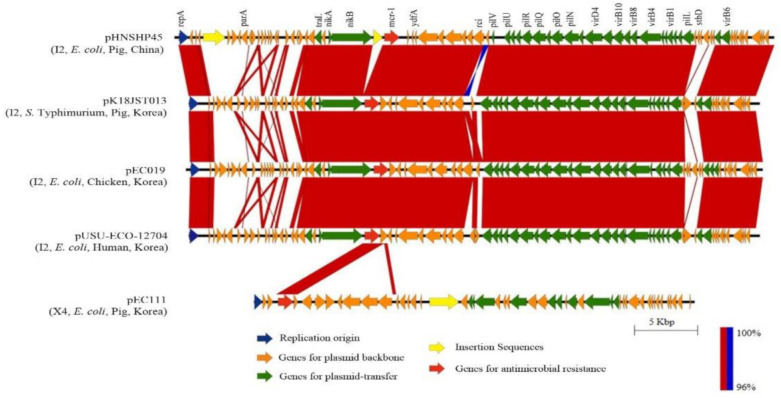
Comparative analyses of the *mcr-1*-carrying pK18JST013 from *Salmonella* Typhimurium with *mcr-1* carrying plasmids in *E. coli* strains isolated from food animals and humans. The sequence of each plasmid (Table S1) was aligned using Blastn (v 2.8.1) and compared using EasyFig (v.2.2.3). Highly conserved regions with normal alignment are indicated in red, and regions with inverted alignment are indicated in blue.

The *mcr*-*1* carrying plasmid (pK18JST013) from this study was compared with those of previously reported strains (Table S1); pK18JST013 was clustered with the plasmid pCFSA664-3 that was detected in *S*. Indiana from chicken in China (Figure 1); pK18JST013 is also related to other *mcr-1*-carrying plasmids detected in *E. coli* and *S*. Typhimurium from humans and food animals in Korea and other countries. Additionally, the *mcr*-*1* carrying plasmid IncI2 pK18JST013 (60,864 bp) had a substantial sequence homology (>98% sequence identity) compared to other IncI2 plasmids. Indeed, insertion sequences were missing upstream of the *mcr-1* gene compared to the original *mcr-1* carrying plasmid from China (pHNSHP45) (Figure 2). The low genetic variability observed among the *mcr*-*1*-carrying plasmids indicates no major evolutionary divergences [52]. Thus, the *mcr*-*1* carrying plasmid might be transmitted to *Salmonella* from other *mcr*-*1*-positive bacteria such as *E*. *coli* which co-exist in gastrointestinal tract food animals [40].

In conclusion, although colistin-resistant *Salmonella* remains rare in food animals in Korea, the detection *mcr*-*1* gene in virulent *S*. Typhimurium strain imposes the greatest awareness. This is the first report of *mcr*-*1*-carrying *S*. Typhimurium in Korea, indicating a recent introduction of this clone into the pig husbandry. This finding emphasizes the role of food animals as potential reservoirs of *mcr*-carrying *S*. Typhimurium ST19.

## Figures and Tables

**Table 1:** Prevalence of colistin resistance in *Salmonella* serogroups isolated from healthy cattle, chickens, and pigs in Korea between 2010 and 2018.

Serogroups	Prevalence (%) (no. of Resistance/no of Tested)
B	0.1 (1/867)
C1	0 (0/996)
C2–C3	0 (0/402)
D1	56.1 (274/488)
E1	0 (0/125)
E2	0 (0/1)
E4	0 (0/74)
G	0 (0/1)
H	0 (0/1)
K	0 (0/1)
L	0 (0/1)
M	0 (0/1)
NT	3.3 (2/60)
Total	9.2 (277/3018)

Abbreviation: NT, Unidentified

**Table 2:** Characteristics of the *mcr*-*1*-carrying *S*. Typhimurium.

Isolate	Source	Isolation Year	Resistance Pattern of Donor	Sequence Type	Conjugation Efficiency	Resistance Pattern of Recipient	Replicon Type of Transconjugant	Virulence Factors
18-A02-013	Healthy Pig	2018	AMP CHL GEN STR FIS TET	ST19	3.0 ± 0.8 × 10^−3^	COL	I2	*spiA, pagC, msgA, invA, sipB, prgH, spaN, orgA, tolC, lpfC, sifA, sopB, iroN, sitC*

Abbreviations: AMP, Ampicillin; CHL, chloramphenicol; COL, colistin; GEN, gentamicin; STR, streptomycin; FIS, sulfisoxazole; TET, tetracycline.

## Data Availability

The datasets presented in this study have been deposited in the Sequence Read Archive of the National Center for Biotechnology Information (NCBI) (accession number: CP065423).

## Supplementary Materials

The following are available online at https://www.mdpi.com/2076-2607/9/2/398/s1. Table S1. Lists of plasmids identified in *E*. *coli* and *Salmonella* isolates recovered from humans and food animals. Table S2. Prevalence of colistin resistance in *Salmonella* Serogroup B and D isolated from cattle, chickens, and pigs in Korea between 2010 and 2018.
