# Supplementary material for: Mobile Colistin Resistance Gene mcr-1 Detected on an IncI2 Plasmid in Salmonella Typhimurium Sequence Type 19 from a Healthy Pig in South Korea
Source: Microorganisms. 2021 Feb 15;9(2):398. doi: 10.3390/microorganisms9020398 (PMC7919004; doi:10.3390/microorganisms9020398)
Supplement: Supplementary file 1 [file microorganisms-09-00398-s001.pdf]

Table S1. Lists of plasmids identified in *E. coli* and *Salmonella* isolates recovered from humans and food animals.

Plasmids	GenBank Accession Number	Plasmid Replicon Type	Bacterial Species	Host	Country	References
**pHNSHP45**	KP347127	I2	*E. coli*	Pig	China	[1]
**pEC019**	KY471145	I2	*E. coli*	Chicken	Korea	[2]
**pEC111**	KY471146	IncX4	*E. coli*	Pig	Korea	[2]
**pEC006**	KY471144	I2	*E. coli*	Chicken	Korea	[2]
**pCFSA664-3**	CP033355	I2	*S.* Indiana	Chicken	China	[3]
**pCFSA244-2**	CP033254	I2	*S.* Typhimurium	Pork	China	[3]
**pCREC-527-4**	KY657476	I2	*E. coli*	Human	Korea	[4]
**pUSU-ECO-12704**	KY657478	I2	*E. coli*	Human	Korea	[4]
**pS3**	PRJEB12529	I2	*S.* Typhimurium	Pig	UK	[5]
**pK18JST013**	CP065423	I2	*S.* Typhimurium	Pig	Korea	This study

Table S2. Prevalence of colistin resistance in *Salmonella* Serogroup B and D isolated from cattle, chickens, and pigs in Korea between 2010 and 2018.

Serogroup	Serotype	No. of isolates	COL-R (%) (No.)
B	Typhimurium	515	0.2 (1)
	4,[5],12:i:-	196	0 (0)
	Agona	59	0 (0)
	Schwarzengrund	28	0 (0)
	Derby	27	0 (0)
	Reading	11	0 (0)
	Kingstone	5	0 (0)
	Lagos	4	0 (0)
	Bredeney	3	0 (0)
	Indiana	3	0 (0)
	Heidelberg	3	0 (0)
	Kaapstad	2	0 (0)
	Lagos	2	0 (0)
	Saintpaul	2	0 (0)
	Stanley	2	0 (0)
	Clackamas	1	0 (0)
	Panama	1	0 (0)
	Brandenberg	1	0 (0)
	Reading 4	1	0 (0)
	Unidentified	1	0 (0)
	Subtotal	867	0.1 (1)
D	Enteritidis	321	49.2 (158)
	Gallinarum	125	92.8 (116)
	Panama	22	0 (0)
	Eschberg	1	0 (0)
	Gueuletapee	1	0 (0)
	Yellowknife	1	0 (0)
	Berta	1	0 (0)
	Unidentified	16	0 (0)
	Subtotal	488	56.1 (274)
Unidentified	Unidentified	60	3.3 (2)
Total		3018	9.2 (277)

COL-R, Colistin resistance

References

1. Grégoire, N.; Aranzana-Climent, V.; Magréault, S.; Marchand, S.; Couet, W. Clinical Pharmacokinetics and pharmacodynamics of colistin. *Clin. Pharmacokinet.* **2017**, *56*, 1441–1460.
2. Lee, J.Y.; Lim, S.K.; Moon, Y.C.; Shin, J.; Ko, K.S. Whole sequences and characteristics of *mcr-1-* harboring plasmids of *Escherichia coli* strains isolated from livestock in South Korea. *Microb. Drug. Resist*. **2018**, *24*, 489–492.
3. Hu, Y.; Fanning, D.; Gan, X.; Liu, C.; Nguyen, S.; Wang, M.; Wang, W.; Jiang, T.; Xu, J.; Li, F. D. *Salmonella* harboring the *mcr-1* gene isolated from food in China between 2012 and 2016. *J. Antimicrob. Chemother.* **2019**, *74*, 826–820.
4. Yoon, E.J.; Hong, J.S.; Yang, J.W.; Lee, K.J.; Lee, H.; Jeong, S.H. Detection of *mcr-1* plasmids in *Enterobacteriaceae* isolates from human specimens: Comparison with those in *Escherichia coli* isolates from livestock in Korea. *Ann. Lab. Med.* **2018**, *38*, 555–562.
5. Anjum, M.F.; Duggett, N.A.; AbuOun, M.; Randall, L.; Nunez-Garcia, J.; Ellis, R.J.; Rogers, J.; Horton, R.; Brena, C.; Williamson, S.; et al. Colistin resistance in *Salmonella* and *Escherichia coli* isolates from a pig farm in Great Britain. *J. Antimicrob. Chemother.* **2016**, 71, 2306–2301.
